# Psychoeducation of Parents of Children with Autism Spectrum Disorders

**DOI:** 10.1192/j.eurpsy.2022.1127

**Published:** 2022-09-01

**Authors:** M. Ivanov, O. Bogacheva, N. Simashkova

**Affiliations:** 1 Federal State Budgetary Scientific Institution, Department Of Child Psychiatry, Moscow, Russian Federation; 2 Mental Health Research Centre, Department Of Childhood Psychiatry, Moscow, Russian Federation

**Keywords:** Autism Spectrum Disorders, psychoeducation, Family, Parental Attitude

## Abstract

**Introduction:**

The family of a child with a mental illness is a significant source for his support in harmonizing his development and achieving successful socialization.

**Objectives:**

The objective of the survey is to develop a psychoeducational program for parents.

**Methods:**

Questionnaire “Parental attitude to children’s illnesses” (V.E. Kagan, I.P. Zhuravleva) Parents of 39 (22 mothers and 17 fathers) children aged from 3 to 6 with ASD - autism spectrum disorders (F84.01; F84.02; F84.11).

**Results:**

Parents of children with ASD often do not realize the morbid nature of changes in the children`s behavior and interpret them as spontaneity, pamperedness or even giftedness. Most parents underestimated the doctor’s recommendations for compliance with the treatment regime. Taking into account the parents` complaints and the difficulties of understanding the child`s problems, a psychoeducational course was developed, including 7 sessions: 1. acquaintance; 2. the concept of ASD, etiological factors, features of manifestation; 3. the role of the family in the treatment and rehabilitation process; 4. development of mental functions in children with ASD; 5. emotional development of children with ASD; formation of communication skills and social adaptation; 6. training organization and correctional and developmental classes for children with ASD; 7. summing up. The psychoeducational course is carried out in the form of group thematic seminars 7 meetings once a week for 1.5-2 hours. After completing the course, some families remain on individual psychological follow-up.

**Conclusions:**

Completing a psychoeducational course makes it possible to fill the lack of information regarding the disease and treatment tactics, increases compliance and harmonizes parent-child relationships.

**Disclosure:**

No significant relationships.

